# Transverse spin relaxation and diffusion-constant measurements of spin-polarized ^129^Xe nuclei in the presence of a magnetic field gradient

**DOI:** 10.1038/srep24122

**Published:** 2016-04-06

**Authors:** Xiaohu Liu, Chang Chen, Tianliang Qu, Kaiyong Yang, Hui Luo

**Affiliations:** 1 Department of Opto-electric Science and Engineering, National University of Defence Technology, Changsha 410073, China

## Abstract

The presence of a magnetic field gradient in a sample cell containing spin-polarized ^129^Xe atoms will cause an increased relaxation rate. We measured the transverse spin relaxation time of ^129^Xe verse the applied magnetic field gradient and the cell temperature. We then compared the different transverse spin relaxation behavior of dual isotopes of xenon (^129^Xe and ^131^Xe) due to magnetic field gradient in the same cell. The experiment results show the residual magnetic field gradient can be measured and compensated by applying a negative magnetic gradient in the sample cell. The transverse spin relaxation time of ^129^Xe could be increased 2–7 times longer when applying an appropriate magnetic field gradient. The experiment results can also be used to determine the diffusion constant of ^129^Xe in H_2_ and N_2_ to be 0.4 ± 0.26 cm^2^/sec and 0.12 ± 0.02 cm^2^/sec. The results are close with theoretical calculation.

The number and variety of applications of noble gases, particularly ^3^He and ^129^Xe, polarized through spin-exchange optical pumping^1^ have grown rapidly over the past few years. Some examples are neutron polarization^2^, studies of surface interactions^3^, magnetic resonance imaging of lungs and other organs of the human body^4^, precision measurements^5^ and quantum computation^6^. All these applications require that the highly non-equilibrium polarizations of the noble gas nuclei be long lived. However, interactions of the polarized noble gas nuclei with alkali atoms, surfaces and the residual magnetic field gradient in the atomic cell can cause rapid relaxation. It’s important to understand these mechanisms and compensate the residual magnetic field gradient to eliminate the influence of the magnetic field gradient on the spin relaxation time for large variety of experiments using polarized noble gases.

The relaxation due to diffusion in free space was first solved by Torrey^7^. He introduced a diffusion term into the Bloch equation applied to the bulk magnetization of a sample containing many spins. R.L. Gamblin and L.D. Schearer first noticed and studied the effects of hyperpolarized ^3^He gas phase relaxation by diffusion through magnetic field gradients within an applied magnetic field^8^,^9^. J. Kestin studied the thermodynamic and transport properties of the five noble gases^10^. R.H. Acosta then did measurements on hyperpolarized ^3^He and ^129^Xe in the presence of a magnetic field gradient^11^. Cates analyzed the spin relaxation of a gas in the presence of a uniform gradient using a perturbation theory approach^12^. Happer’s group in the Princeton University conducted systematic theoretical and experimental research on the mechanism of the influence of the magnetic field gradient on the gas atoms spin relaxation. They designed experiments which can effectively reduce the influence of the error factors. The theory they have developed can very well explain the experiment results about the atomic spin relaxation in the presence of magnetic field gradient. They also measured the diffusion constant of Xe in 760 Torr of either He or N_2_ at 80 °C to be 0.791 ± 0.032 and 0.21 ± 0.03 cm^2^/sec^12^,^13^,^14^. The origin of the relaxation mechanism is the loss of phase coherence of the atoms due to fluctuating magnetic field, which is felt by the atoms as they diffuse throughout the cell. McGregor applied the Redfield theory to this problem and yielded theoretical expressions for transverse relaxation rate of a spin-polarized gas due to a magnetic field gradient. The expression agrees with that of Cates in the high-pressure limit^15^. The work of Golub shows that these two approaches are identical^16^. Recently, searches for new Parity and Time reversal violating forces mediated by the unobserved axion in connection with this subject drew people’s new attention^17^.

In the Nuclear Magnetic Resonance (NMR) gyroscope system, magnetic shield is commonly used to shield the external field. Magnetic shielding ability is limited by conditions of magnetic shielding system. The assembling process and environmental vibration may cause the positions of the vapor cells offset relative to the symmetry center of the coil system. Thus the vapor cell may feel a magnetic field gradient in the NMRG system. Running the NMR gyroscope with real time closed loop control of all three magnetic field directions can suppresses low frequency magnetic field noise including Johnson noise from the magnetic shields, but the compensation of the residual magnetic field was barely mentioned in some recent NMRG papers^18^,^19^.

In this paper, we used the magnetic field gradient coils to measure and compensate the residual magnetic field gradient in the NMRG system. We measured the transverse spin relaxation time of ^129^Xe at different temperature. Both the binary collisions and the three-body collisions of van der Waals molecules contributed to the relaxation rate of ^129^Xe in our system. We first measured the transverse spin relaxation times of dual isotopes of xenon (^129^Xe and ^131^Xe) contained in the same cell versus the applied magnetic field gradient. The different magnetic field gradient dependences of the two isotopes agreed with the theoretical calculation. The experimental results show that the residual magnetic field gradient can be measured and compensated by applying the magnetic gradient in the NMRG vapor cell, besides, spin relaxation in inhomogeneous magnetic fields could be used to determine the diffusion constant of Xe in 70 Torr of N_2_ and 20 Torr of H_2_.The results are close with theoretical calculation from the formula proposed by Fuller^20^.

## Results

The transverse relaxation rate of noble gas atoms in a NMRG cell can be expressed as^21^,^22^,^23^,^24^,^25^:


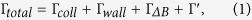


where Γ_*coll*_ is the relaxation in alkali-metal collisions, Γ_*wall*_ is the relaxation due to collisions with the cell wall surface, Γ_Δ*B*_ is the relaxation due to magnetic-field inhomogeneities. Γ′ is the gas-phase relaxation due to self-collisions of noble gas atoms and can be negligible compared with other influences in our system.

For binary collisions, the spin-relaxation rate can be written simply as the product of the alkali density *n*_*Rb*_, a spin-exchange cross-section *σ*, and mean relative thermal velocity 
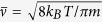
, where m is the reduced mass in the alkali-noble gas collision, *k*_*B*_ is the Boltzmann’s constant, T is the cell temperature^21^.

The relaxation rate from the formation of van der Waals molecules is inversely proportional to the noble gas density. At high buffer-gas density, the lifetime of the weakly bound van der Waals molecules can be reduced to the point that the molecules do not live long enough for spin exchange to occur efficiently, and the spin-exchange rate due to binary collisions can exceed the spin-exchange rate due to van der Waals molecules. Combing the effects of binary collisions and van der Waals molecule formation, the relaxation rate Γ_*coll*_ is^22^,^23^:


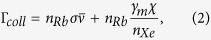


where *n*_*Xe*_ is the noble gas density, the rate *γ*_*m*_ is a constant and has been measured by G. D. Cate. The factor *χ* is nearly a constant and depends on the nuclear spin and relative abundance of ^87^Rb.

For the case of ^129^Xe, silicone coatings are known to extend wall relaxation times substantially from tens of seconds to tens of minutes^25^. Steven R. Breeze showed that spin-lattice relaxation times (T_1_) could even be extended for several hours under the right conditions (cell coating, cell size, homogeneous magnetic field, and optimal optical pumping conditions)^26^,^27^. In our spin-exchange optical pumping experiments, the vapor cell has no coatings, so the wall relaxation times may be tens of seconds.

The relational expression of the magnetic field gradient and the transverse spin relaxation rate of noble gas atoms can be expressed as^12^,^14^:


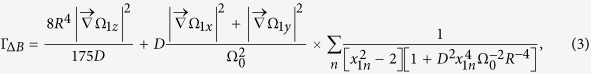


where D is the diffusion constant of noble gas atoms in the atomic cell. To calculate the diffusion constant of two kinds of gas atoms, the formula proposed by Fuller^20^ can be used.


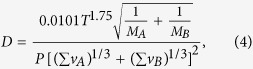


where P is the total pressure of the two gases, T is the temperature, M_A_ and M_B_ is the molecular weight of binary gas A and B. 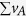 and 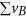 is the molecular diffusion volume. We referred in^20^ that 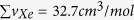, 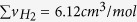 and 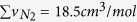. The results of theoretical calculation: the diffusion constant of Xe gas in the Xe gas is 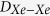 = 0.064 cm^2^/sec, the diffusion constant of Xe gas in the N_2_ gas is 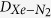 = 0.13 cm^2^/sec, the diffusion constant of ^129^Xe gas in the H_2_ gas is 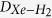 = 0.6 cm^2^/sec.

R is the radius of the vapor cell, x_1n_ is the spatial frequency coefficients related to the Bessel function. Ω_0_ = *γB*_0_ is the precession frequency of the noble gas atomic spins. γ is the gyromagnetic ratio of the noble gas atom, B_0_ is the static field.


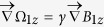
, 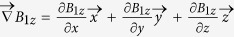, where B_1_ is the gradient magnetic field applied. We added a pair of gradient magnetic field coils to the vapor cell, the axis of the coils was along the direction of the static field. The symmetry center of the coils was consistent with the atomic cell center. Thus, the magnetic field produced by the gradient magnetic field coils can be cancelled at the atomic cell center. The magnetic field gradient produced by the gradient coils at the atomic cell center is:


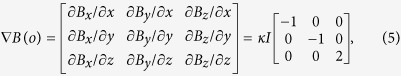


where I is the intensity of the electric current in the coils, κ is the coil calibration constants, the expression of κ is:


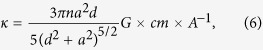


where n is the number of turns of the coils, a is the radius of the coils, d is the distance the coils are separated by. When the radius of the coils is much larger than the radius of the atomic cell, we can regard that equation (6) is independent of the position within the cell.

Equation (3) can be written in the form of:


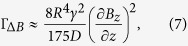


When the vapor cell is maintained at a static temperature, the total transverse relaxation rate of noble gas atoms can be expressed as:


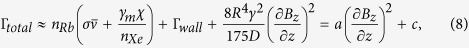


It should be noted that the magnetic field gradient in this paper is referred to ∂*B*_*z*_/∂*z*. When we change the intensity of the electric current flowing in the gradient coils, we can change the magnetic field gradient in the vapor cell. Combining with the theoretical relationship of the magnetic field gradient ∂*B*_*z*_/∂*z* in the vapor cell and the transverse spin relaxation rate of noble gas atoms, we can get transverse spin relaxation time of ^129^Xe verse the applied magnetic field gradient and determine the diffusion constant of ^129^Xe in N_2_ and H_2_.

The sample cell is made of Pyrex glass which is spherical in shape with diameter of about 1cm. Cell 1~4 contains a few milligrams of ^87^Rb metal, 2 Torr of ^129^Xe, 70 Torr of N_2_ and 20 Torr of H_2_ to quench the excited state of ^87^Rb atoms. Cell 5 contains 2 Torr of ^129^Xe, 8 Torr of ^131^Xe, 70 Torr of N_2_ and 20 Torr of H_2_. Schematic of the apparatus used for the NMR shifts measurements is almost the same with Fig. 1 in ref. 28. A pump beam emitted from a high power diode laser is tuned to the center of the ^87^Rb D1 line. A linearly polarized probe beam tuned to the center of the ^87^Rb D1 line transmits through the center of our cell. It will lead to a Faraday rotation angle *θ* in the magnetic field when linearly polarized light propagates through the cell. The outgoing light transmitting through a *λ*/2 plate and a Wollaston prism and will split into separate components of vertically and horizontally linearly polarized light. The intensities of the two linearly polarized lights detected by the balanced photodetector are:


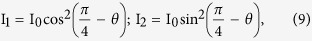



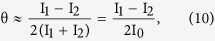


The Faraday rotation angle *θ* can be determined by finding the difference of the two component beam intensities. Then we can deduce the magnetic field induced by the precession of ^129^Xe in the x-y plane.

**Figure 1:**
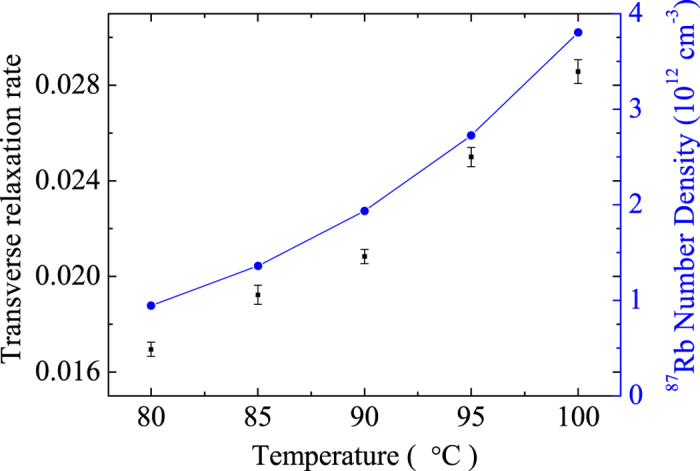
The transverse spin relaxation rate of ^129^Xe and the ^87^Rb number density versus the temperature.

We first measured the transverse spin lifetime of ^129^Xe using Free Induction Decay (FID) method. We used a *π*/2 pulse to drive up the ^129^Xe precession and then allow the coherence to decay naturally. We fitted the decaying curve with a simple exponential curve *f* = *Ae*^(−*T*/*τ*). Then the transverse spin lifetime could be extracted from the fitting solution. We measured the transverse spin lifetime of ^129^Xe and the ^87^Rb number density in cell 1 from 80 °C to 100 °C in intervals (80, 85, 90, 95, 100) where the temperature was allowed to equilibrate before taking measurements. We then got the transverse spin relaxation lifetime rate and the ^87^Rb number density versus the temperature as shown in Fig. 1. We found the transverse spin relaxation lifetime rate and the ^87^Rb number density increased with the temperature. The transverse spin relaxation rate nearly linearly corresponded to the ^87^Rb number density, which was consistent with Eq. (2). We could then deduce the transverse spin relaxation rate of ^129^Xe due to wall collisions, magnetic-field inhomogeneity and other relaxation mechanisms that did not depend on the ^87^Rb number density.

**Figure 2:**
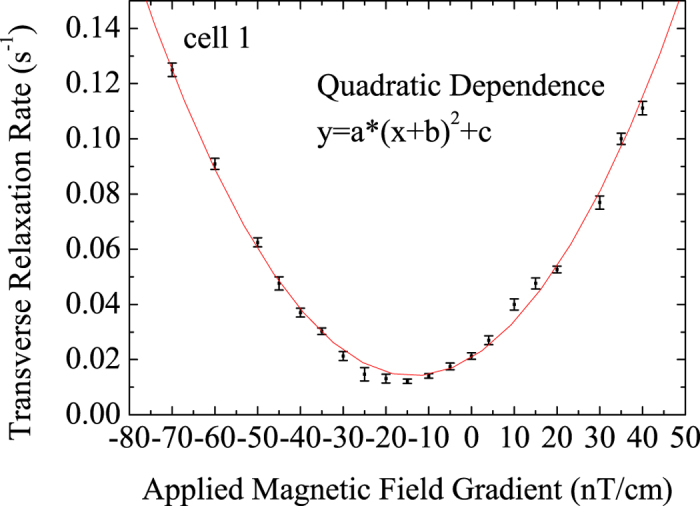
The influence of the magnetic field gradient on the transverse relaxation rate of ^129^Xe.

We measured the transverse relaxation rate of ^129^Xe in cell 1 versus the applied magnetic field gradient ∂*B*_*z*_/∂*z* = 2*κI*. It should be noticed that, the step length of changing the electric current input into the gradient coils should be as short as possible. According to the theoretical relationship expression of the spin relaxation time and the magnetic field gradient, which was consistent with Eq. (8), we can fit the measurement results of the relationship of the magnetic field gradient and the spin relaxation rate by quadratic function type through least square method. As shown in Fig. 2, the data showed that the relaxation rate of ^129^Xe as a function of applied magnetic field gradient was in quadratic dependence. The center of the quadratic curve where the transverse spin relaxation rate reached minimum deviated from the zero point. This meant that the residual magnetic field gradient in the magnetic shields directed the same orientation of the static magnetic field.

**Figure 3:**
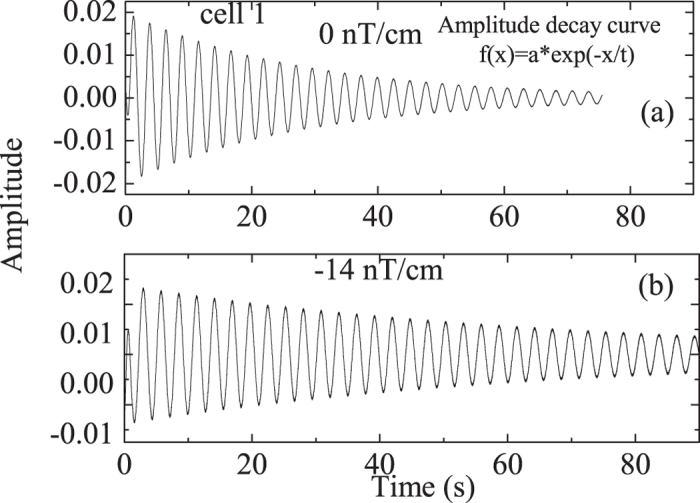
Relaxation time of ^129^Xe. (**a**) Without the compensating magnetic field gradient; (**b**): with the compensating magnetic field gradient.

We can compensate the residual magnetic field gradient by applying a negative magnetic gradient in the sample cell. As shown in Fig. 3, when magnetic field gradient is not applied, the residual magnetic field gradient is not compensated, the transverse spin relaxation time of ^129^Xe was about 43s. When applied the reverse magnetic field gradient −14 nT/cm to compensate the residual magnetic field gradient, the transverse spin relaxation time of ^129^Xe was about 68s. The residual magnetic field gradient can be measured and compensated by using the magnetic field gradient coils in the NMRG vapor cell to extend spin relaxation time of the noble gas atoms. We measured cell 1~5 and found the transverse relaxation time of ^129^Xe could be increased 2–7 times longer when applying an appropriate magnetic field gradient. The results were showed in Table 1. T_2_ is the transverse relaxation time and T’_2_ is the transverse relaxation time with the residual magnetic field gradient compensated. ∂*B*_*z*_/∂*z* is the compensating magnetic field gradient.

The transverse relaxation times of ^129^Xe and the magnetic field gradient felt by the cells have a great disparity. When we changed cells in the experimental system, the cells had different offsets relative to the symmetry center of the coil system and thus felt different magnetic field. Another reason was the difference in the cell fabrication (cell shape, inner surface, etc), which could have influence on the relaxation time. The relaxation times of ^129^Xe in cell 5 were less than that of cell 1~4 with or without the residual magnetic field gradient compensated. The reason for this may be the added 8 Torr of ^131^Xe, whose polarization could affect the polarization and relaxation rate of ^129^Xe. No one has really studied this issue very carefully, so it could well be that the two isotopes affect each other relaxation rates, especially at high gas pressures where the increasing fraction of ^129^Xe and ^131^Xe molecules may help the isotopes to interact. To study this, more measurements should be done without the added complexity of ^87^Rb atoms and optical pumping.

**Figure 4:**
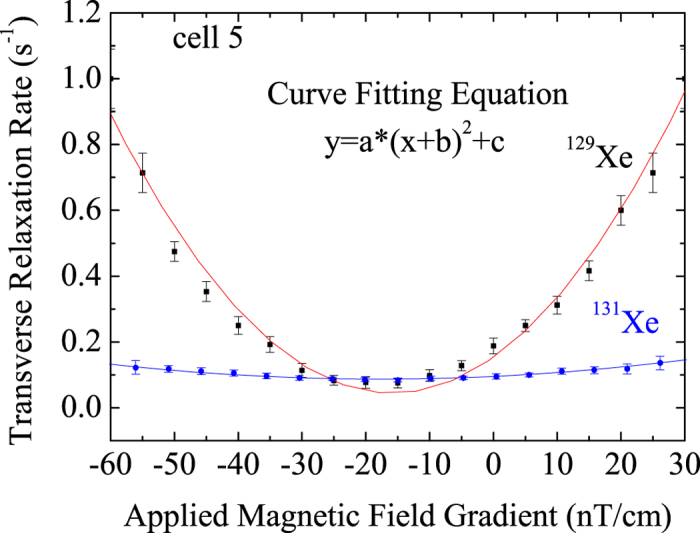
The transverse relaxation rates of ^129^Xe and ^131^Xe versus the magnetic field gradient.

The magnetic field gradient has much less influence on the transverse relaxation time of ^131^Xe increased than that of ^129^Xe from the data in cell 5. We compared the transverse relaxation rate of ^129^Xe and ^131^Xe versus the applied magnetic field gradient as shown in Fig. 4. The relaxation rates of both ^129^Xe and ^131^Xe as a function of applied magnetic field gradient were in quadratic dependence. We fitted the measurement results of the relationship of the magnetic field gradient and the relaxation rates by quadratic function type. The curve of ^131^Xe was flatter than that of ^129^Xe as the magnetic field gradient changed. The fitting results for ^129^Xe and ^131^Xe are *a*_129_/*a*_131_ ≈ 13.2. This can be explained by the different gyromagnetic ratios of the two isotopes, which is used to calculate the value of a in Eq. (8).


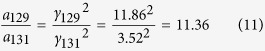


The fitting results are close with the theoretical calculation.

**Figure 5:**
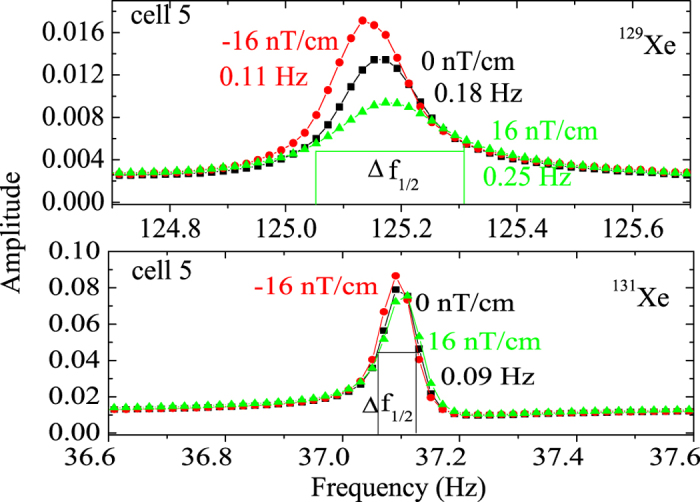
The NMR signals of ^129^Xe and ^131^Xe versus the applied magnetic field gradient.

When applying magnetic field gradient using the magnetic gradient coils, we found the nuclear magnetic resonance frequency of ^129^Xe and ^131^Xe would shift with the applied magnetic field gradient. The frequency shifts due to magnetic field gradient had relation to the position the cell centered relative to the magnetic field gradient coils^29^,^30^,^31^,^32^,^33^,^34^. As shown in Fig. 5, the NMR frequency of ^129^Xe decreased about 0.02 Hz when applied the reverse magnetic field gradient −16 nT/cm while the NMR frequency of ^131^Xe decreased about 0.006 Hz. The ^131^Xe’s electric quadrupole interaction of the nuclei with the surface is averaged to zero because of the spherical shape of the sample cell and no quadrupole splitting is observed. The frequency shifts caused by the applied magnetic field gradient can be used as error signal to demarcate the position the cell centered relative to the magnetic field gradient coils in the system.

When the applied magnetic field gradient was −16 nT/cm, 0 nT/cm and 16 nT/cm, the linewidth (Full Width at Half Maximum, FWHM) of ^129^Xe was about 0.11 Hz, 0.18 Hz and 0.25 Hz while that of ^131^Xe nearly does not change. The magnetic field gradient has much less influence on the transverse relaxation time of ^131^Xe than ^129^Xe, which is correlated with the linewidth:


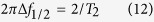


The magnetic field created by the precessing ^129^Xe magnetization vector, and sensed by the rubidium atoms, can be estimated as


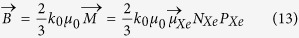


where *k*_0_ is the spin-exchange enhancement factor, *μ*_0_ is the permeability of free space, 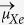 is the magnetic moment of the ^129^Xe nucleus, *N*_*Xe*_ is the xenon number density, and *P*_*Xe*_ is the fraction of the noble gases. The relaxation due to magnetic-field inhomogeneity decrease when applied the appropriate reverse magnetic field gradient and thus the degree of polarization of the ^129^Xe atoms increases. We could also see that the amplitude of the ^129^Xe changes larger than the amplitude of ^131^Xe, which agreed with the theory.

Comparing the value theoretically calculated and the value experimentally obtained, we can determine the diffusion constant of ^129^Xe in N_2_ and H_2_. The sample cell 1 contains 70 Torr of N_2_ and 20 Torr of H_2_. The diffusion constant of noble gas atoms in the atomic cell be expressed as:


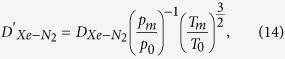



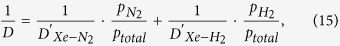


where *p*_*m*_, *T*_*m*_ is the pressure and temperature in our experimental condition, *p*_0_, *T*_0_ is the pressure and temperature in standard condition, 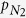 is the pressure of N_2_, 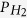 is the pressure of H_2_, *P*_*total*_ is the total pressure of the gases in the vapor cell. According to the Eqs (8) and (15), the value of a calculated from 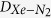 = 0.13 cm^2^/sec and 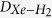 = 0.6 cm^2^/sec was 3.224*10^−5^. The fitting result in Fig. 2 is a = (3.41 ± 0.17)*10^−5^. We can determine the diffusion constant of ^129^Xe in N_2_ and H_2_ in cell 1 from the fitting result: 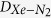 = 0.124 ± 0.006 cm^2^/sec, 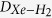 = 0.38 ± 0.22 cm^2^/sec. Averaging the values of a obtained from the fitting results of all the measured cells, the diffusion constant of ^129^Xe in N_2_ and H_2_ is 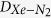 = 0.12 ± 0.02 cm^2^/sec, 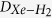 = 0.4 ± 0.26 cm^2^/sec. The experimental results are close with the theoretical calculation. The results given by K. C. Hasson were at the temperature of 80 °C while our values were converted to the room temperature. They measured the spin relaxation rate in the rotating coordinate system. The pump laser light was blocked after the cell was polarized for about 3–5 minutes when they measured the relaxation time^14^. Our relaxation time was measured in the laboratory system and the pump laser was always illuminating the cell to maintain a large number of ^87^Rb atoms polarized.

## Discussion

We analyzed the relaxation mechanisms in our NMRG cell. We found both the binary collisions and the three-body collisions of van der Waals molecules contributed to the relaxation rate of ^129^Xe in our system. We used the magnetic gradient coils to measure and compensate the residual magnetic field gradient in the NMRG system. When applied the appropriate reverse magnetic field gradient, the transverse spin relaxation time of both ^129^Xe and ^131^Xe could be increased. We compared the frequency shifts and relaxation rates due to the magnetic field gradient of ^129^Xe and ^131^Xe. The different dependence on magnetic field gradient of the dual isotopes could be well described by theoretical calculation. We can use the dual isotope to measure the magnetic field gradient and to demarcate the position the cell centered relative to the magnetic field gradient coils in the system more exactly. Comparing the value of a in Eq. (8) from theoretically calculated and from experimental curve fitting result, we determined the diffusion constant of ^129^Xe in N_2_ and H_2_. The measured diffusion constant of ^129^Xe in N_2_ is near the theoretical calculation, but the measured diffusion constant of ^129^Xe in H_2_ has a relatively large error range. The reason is that most of the buffer gas contained in the cell is N_2_ and N_2_ dominates the diffusion of ^129^Xe.

In summary, we used the magnetic gradient coils to compensate the residual magnetic field gradient and demonstrated the transverse spin relaxation time of both ^129^Xe and ^131^Xe could be increased in the NMRG system. We compared the different behavior of dual isotopes due to magnetic field gradient in the same cell, the results agreed well with the theory. We measured the diffusion constant of ^129^Xe in N_2_ and H_2_. The results were close with theoretical calculation. We found the added ^131^Xe could affect the polarization and relaxation rate of ^129^Xe. To research this, we should do more NMR measurements without the added complexity of ^87^Rb atoms and optical pumping.

## Methods

The cell is heated by flowing hot air and is shielded by a set of five cylindrical magnetic shields. There are two-axis field coils providing control of the magnetic field inside the shields. The structure of the static coils and gradient coils meet the conditions: the distance coils are separated by the radius of the coils = 76 mm. Each of the pair of the static coils has 10 turns, producing a stable and highly homogeneous static magnetic field of 9.9 μT; each of the pair of the gradient coils has 5 turns, producing magnetic field gradient of 9.4 nT/(cm · mA) by inputting direct electric current to the coils. The material of the coils is copper, which has low impedance. The power of the pump laser is a few tens of milliwatts to polarize a large number of ^87^Rb atoms.

## Additional Information

**How to cite this article**: Liu, X. *et al.* Transverse spin relaxation and diffusion-constant measurements of spin-polarized ^129^Xe nuclei in the presence of a magnetic field gradient. *Sci. Rep.* **6**, 24122; doi: 10.1038/srep24122 (2016).

## Figures and Tables

**Table 1:** Transverse Relaxation times of Xenon in cell 1~5.

Cell	T_2_(s)	T’_2_(s)	∂*B*/∂*z* (nT/cm)
1 ^129^Xe	43 ± 1	68 ± 0.6	−14
2 ^129^Xe	10 ± 1.2	28 ± 0.8	−23.5
3 ^129^Xe	13 ± 0.8	44 ± 1	−30
4 ^129^Xe	9.8 ± 1	72.6 ± 1.2	−44.3
5 ^129^Xe	6 ± 1.2	13.2 ± 0.6	−16
^131^Xe	10 ± 0.8	12 ± 0.4	−16

T_2_ is the transverse relaxation time. T’_2_ is the transverse relaxation time with the residual magnetic field gradient compensated. ∂*B*/∂*z* is the compensating magnetic field gradient.
